# A rare presentation of a 17-year-old female with primary colon lymphoma, presenting as intussusception: a case report

**DOI:** 10.1097/MS9.0000000000005139

**Published:** 2026-05-14

**Authors:** Mariam A. Uledi, Arapha A. Mvugalo, Hudson C. Nyondo, Jesse J. Kashabano, Alex B. Mashaka, Emmanuel L. Lugina

**Affiliations:** a Department of Clinical Oncology, Muhimbili University of Health and Allied Sciences, Dar es Salaam, Tanzania; b Department of Oncology, Bugando Medical Centre, Mwanza, Tanzania; c Department of Oncology, Benjamin Mkapa Hospital, Dodoma, Tanzania; d Department of Pathology, Muhimbili University of Health and Allied Sciences, Dar es Salaam, Tanzania; e Oncology Department, Ocean Road Cancer Institute, Dar es Salaam, Tanzania

**Keywords:** adolescent, case report, diffuse large B-cell lymphoma, primary colon lymphoma, Tanzania

## Abstract

**Introduction and importance:**

Primary colonic lymphomas are rare malignancies that mainly occur in men in their fifth to seventh decades, accounting for less than 1% of colorectal cancers. Most are B-cell non-Hodgkin lymphomas, with diffuse large B-cell lymphoma (DLBCL) being the most common subtype. Diagnosis is often challenging due to nonspecific symptoms, and management typically relies on systemic immunochemotherapy. Surgery retains a vital role in selected cases, particularly those with complications or localized disease. We present an interesting case of a 17-year-old girl with primary colonic DLBCL presenting with intussusception, who was treated at Ocean Road Cancer Institute in Tanzania.

**Case presentation:**

A 17-year-old female of African origin experienced chronic abdominal pain and a loss of appetite for 6 months. An ileocolic intussusception was diagnosed through a CT scan and intraoperative findings, and a right hemicolectomy was performed. Histopathological examination of the resected specimen revealed DLBCL of the colon, with immunohistochemistry positive for CD20 and CD45, confirming a diagnosis of primary colorectal lymphoma. Later, the patient received systemic chemotherapy with R-CHOP for eight cycles. She is clinically stable and has been under follow-up for the past year.

**Conclusion:**

Primary colonic DLBCL in adolescents is exceedingly rare. This case presents unique features such as chronic abdominal pain and anorexia, which can lead to misdiagnosis due to a broad differential. There is a need to provide knowledge of primary colon lymphoma to ensure early diagnosis and favorable outcomes.

## Introduction

The gastrointestinal tract (GI) is the most common extranodal site involved by lymphoma, accounting for 5–20% of all cases[1]. Primary GI lymphoma, however, constitutes only about 0.2–1.2% of all gastrointestinal malignancies[2]. In pediatrics, primary GI lymphoma is rare and constitutes less than 5% of all pediatric neoplasms[3]. The mean age for primary GI lymphoma is within 5–15 years[4]. The male-to-female ratio is reported to range from 7:1 to 1.8:1[5]. Primary GI lymphoma is usually secondary to widespread nodal diseases. Lymphoma can develop anywhere in the GI tract, but it most frequently affects the stomach, followed by the small intestine and the ileocecal region^[^1,6^]^. The order of involvement is as follows: stomach (65%), small intestine (20–30%), colon (10–20%), and esophagus (1%)[6]. The most common histological subtype is diffuse large B-cell lymphoma (DLBCL); however, other types, such as mucosa-associated lymphoid tissue (MALT) lymphoma, may also occur. GI T-cell lymphomas are rare[7]. Certain histological subtypes have been noted to have a relative predilection for specific sites, such as MALT lymphoma in the stomach, mantle cell lymphoma in the terminal ileum, jejunum, and colon, as well as enteropathy-associated T-cell lymphoma in the jejunum, and follicular lymphoma (FL) in the duodenum, with a geographic variation in its distribution[1].

HIGHLIGHTS

- The primary colon lymphoma in an adolescent female as an addition to the pediatric data on gastrointestinal malignancies.
- This case highlights a rare presentation of primary colon diffuse large B-cell lymphoma in young adults.
- This will enhance the need for awareness and vigilance in clinical practices so that these cases do not go unnoticed, and the importance of being familiar with accurate evaluation and management options for primary colon lymphoma is recognized.
- It is essential to consider lymphoma in the differential diagnosis for patients presenting with chronic abdominal pain.

The GI tract is prone to secondary involvement of lymphoma, as mesenteric or retroperitoneal lymph nodes are common sources of lymphoma that may drain lymphoid tissue from the GI tract[8]. To make the diagnosis of primary GI lymphoma, the standard criteria were introduced by Dawson *et al* in 1961, as outlined in Table 1^[^9^]^.

**Table 1:** Dawson’s diagnostic criteria for primary GI lymphoma.

1	Absence of clinically enlarged lymph nodes on clinical examination
2	Absence of enlarged mediastinal lymph nodes on chest X-ray
3	Normal hematologic lab values on bone marrow biopsy
4	Normal-appearing liver and spleen
5	Only regional lymph nodes are present at the time of laparotomy

The risk factors for GI tract lymphoma include HIV, *Helicobacter pylori* infection, celiac disease, inflammatory bowel disease, *Campylobacter jejuni*, Epstein-Barr virus, hepatitis B virus, human T-cell lymphotropic virus-1, and immunosuppression after solid organ transplantation[1].

Only 0.2% of all malignant tumors arising from the colorectal region are classified as primary colorectal lymphoma (PCL) due to its rare occurrence, with the cecum, ascending colon, and rectum being the most commonly affected areas. The disease predominantly affects men, presenting with abdominal pain, weight loss, a palpable abdominal mass, or lower gastrointestinal bleeding. Obstruction and perforation are relatively rare in patients with PCL. The primary risk factors for PCL are immunosuppression and inflammatory bowel disease; however, the direct causes are not yet fully understood[10]. Standardized treatment for PCL is not well established[11].

PCL is rare among young pediatric patients, particularly in young women, and presents a diagnostic challenge due to varied clinical presentations that overlap with other GI diseases, often leading to misdiagnosis and delayed management and treatment, such as surgery and chemotherapy. Here, we describe a unique case of primary DLBCL in a 17-year-old girl in the ascending colon, presenting with intussusception, and explore its clinicopathological features, diagnostic criteria, management, and prognosis. This case report has been prepared in accordance with the SCARE checklist[12].

## Case presentation

**Figure 1.**
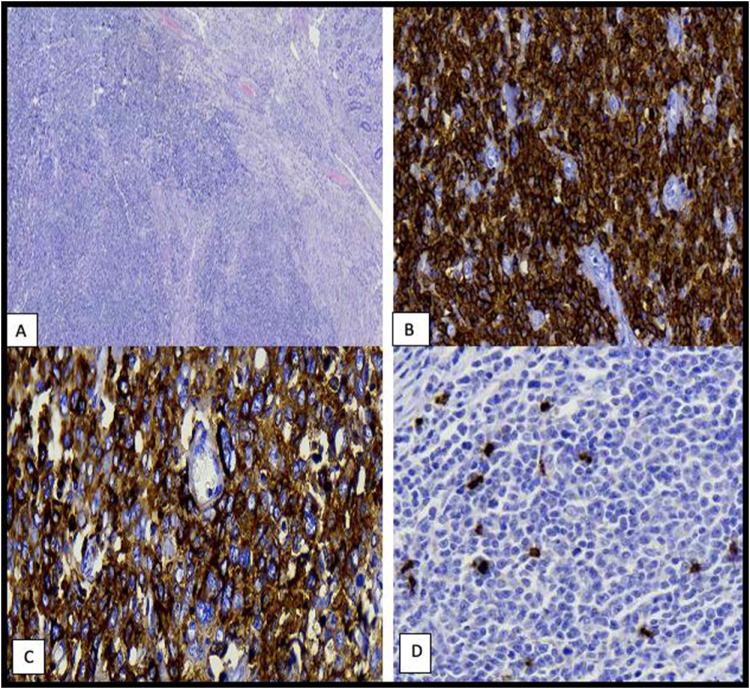
Hematoxylin and eosin-stained and immunohistochemistry sections: shows H&E stained colonic tissue with tumor infiltrate in the stroma in diffuse process composed of discohesive round blue cells (A, ×40 magnification). Immunohistochemistry markers are positive for CD45 (B, ×400 magnification) and CD20 (C, ×400 magnification), while negative for CD3 in tumor cells but rather scattered positivity in infiltrating reactive mature T-cells (D, ×400 magnification).

A 17-year-old female of African origin, who was pursuing ordinary-level secondary education, presented with chronic abdominal pain on the right side, colic in nature, which was on and off and associated with loss of appetite. The pain was not associated with abdominal distension or changes in bowel habits, and it was relieved with the use of painkillers. The abdominal pain persisted for 4 months, preventing her from progressing with her studies. Later on, she was diagnosed with appendicitis and underwent an appendectomy at a peripheral clinic. The abdominal pain persisted and increased despite undergoing an appendectomy a month earlier and was now only alleviated by morphine solution. She underwent esophagogastroduodenoscopy and colonoscopy, which revealed mild gastritis and an ileocecal polyp, respectively; however, we could not retrieve the images. A CT scan of the abdomen with contrast was done and revealed an over-distended stomach containing residual food contents, suggesting gastric outlet obstruction, and a filling defect in the ascending colon. Emergency exploratory laparotomy revealed ileocolic intussusception with an intraluminal ileal polyp, for which a right-sided hemicolectomy was performed. Histopathological examination of the biopsy revealed right-sided primary colon non-Hodgkin’s lymphoma, and immunohistochemistry results showed a cluster of differentiation CD20 and CD45 positive, which confirmed DLBCL with CD3 negative, as shown in Figure 1. Molecular genetic alteration studies, such as translocations [t(14;18), t(3;14)], IgH rearrangements, chromosomal gains [3q (BCL-6/FOXP1), 13q, and 18q], or losses (8p, 9p, and 17p), were not performed due to a lack of resources and financial constraints. A bone marrow aspirate or biopsy was also not performed. She was sent to our facility for oncological management a month after the right hemicolectomy. At our facility, she presented with no complaints. The patient’s guardian denied any prior treatment with radiation, a similar condition among her siblings, or a family history of malignancies. On examination, she was alert, afebrile, and not pale or jaundiced. There was no lower limb edema. Her blood pressure was 120/76 mmHg, her pulse rate was 105 beats per minute, and her temperature was 36.5°C. Her functional status was Eastern Cooperative Oncology Group 0. On local examination, she had a healed extended surgical scar on the abdomen and a right oblique scar, with no organomegaly and no palpable axillary or inguinal lymph nodes. Her Glasgow Coma Scale score was 15/15. Other systems were essentially uneventful. HIV-1 serology was negative; her hemoglobin level was 9.1 g/dl, and her white blood cell count was normal. The hepatitis B and C panels were negative. Additionally, she had a normal level of lactate dehydrogenase (LDH) at 429 IU/l, and her renal and liver function tests were within normal limits. Due to financial constraints, the patient was only able to undergo a chest X-ray and an ultrasound of the abdomen and pelvis, which were normal. Based on the imaging results, using Lugano staging, she was classified as stage II, and using the age-adjusted International Prognostic Index (IPI), she was classified as low risk. After a multidisciplinary team discussion, the patient was initiated on systemic chemotherapy with the R-CHOP regimen. She received rituximab 390 mg intravenously during the first cycle only of R-CHOP due to financial constraints. This sequence of five cycles was in the form of a CHOP regimen (cyclophosphamide 1 g IV, doxorubicin 70 mg IV, vincristine 2 mg IV, and prednisolone 300 mg orally, three times daily, for 5 days). Supportive care included antiemetics and multivitamin supplements. The patient tolerated chemotherapy well, with no significant adverse effects such as diarrhea or vomiting, as confirmed through laboratory workups and performance status. Upon completion of treatment, a reassessment with abdominal and pelvic ultrasound, chest radiography, and serum LDH revealed no abnormalities. Afterward, she was scheduled for outpatient follow-up every 3 months.

**Figure 2.**
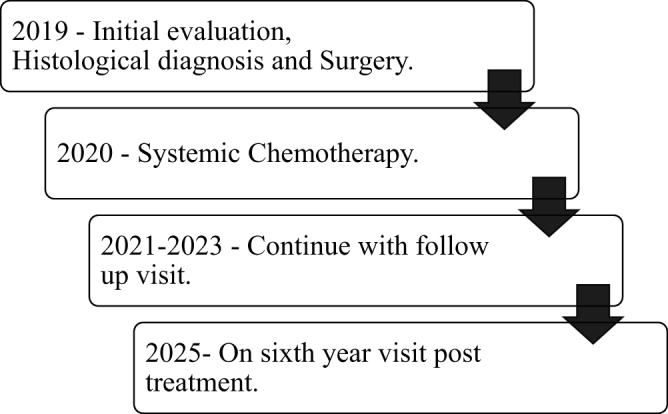
Summary of important events in the treatment circle of the case.

At the time of reporting this case, the patient is 24 years old and has completed her sixth follow-up visit, with no clinical or radiological evidence of tumor recurrence. A summary of the timeline of important clinical events is provided in Figure 2.

## Discussion

We discuss a case of PCL in a 17-year-old female patient. A literature search revealed only five articles on PCL with patients aged less than 30 years (Table 2). To the best of our knowledge, we believe our case is the second youngest female patient worldwide and the first in Africa.

**Table 2:** Reported cases of PCL with an age of less than 30 years.

*Author, year*	*Age*	*Gender*	*Location*	*Histology*
Bandyopadhyay *et al*, 2011^[^12^]^	8	M	Sigmoid colon	Burkitt’s Lymphoma
Jiao *et al*,2014^[^3^]^	16	M	Ascending colon	T-cell Lymphoma
Saka *et al*, 2015^[^13^]^	14	M	Ileocecal junction	DLBCL
Qadri *et al*, 2016^[^14^]^	11	M	Ascending colon	DLBCL
Sangma *et al*,2016^[^15^]^	27	F	Ileocecal region, cecum, and ascending colon	Burkitt’s Lymphoma
Pandey *et al*, 2019^[^16^]^	13	M	Caecum and ascending colon	DLBCL
	20	M	Rectum and rectosigmoid junction	DLBCL

According to Dionigi *et al*, PCL commonly presents with nonspecific symptoms, most frequently abdominal pain (66.8%), anorexia and weight loss (43%), an abdominal palpable mass (41.3%), and bloody stool (23%). Less common presentations include acute abdomen, microcytic anemia, rectal bleeding, and changes in bowel habits[10]. Rare symptoms include bowel perforation, obstruction, and intussusception[3]. Ileocolic intussusception is a common condition in young children, but it is rare in adolescents and adults, and pathological lead points (such as a polyp, Meckel’s diverticulum, or neoplasm) are often identified[13]. The patient in this case report presented with abdominal pain, which was on and off for about 6 months and was not associated with distension or changes in bowel habits. Notably, she presented with intussusception, a rare finding for PCL. Similar findings have been observed by Saka *et al*[14]. Our patient had DLBCL located on the right side of the colon; however, our female patient presented with primary colon lymphoma (PCL) at 17 years of age, which is different from most literature, as it is seen mainly in adults. However, it coincides with the case report done in India, where one of the patients was 13 years of age at diagnosis[15].

The PCL in children/adolescents is rare, and unlike the one in adults, which occurs in the stomach, most of the children's PCL occurs in the small intestine[3]. Clinically, patients present with varied and non-specific symptoms ranging from an abdominal mass to an acute abdominal emergency caused by intussusception, making diagnosis a challenge with a risk of misdiagnosis, as it is difficult to distinguish from other differential diagnoses such as enteritis, inflammatory bowel disease, or leiomyoma[16]. Most of the diagnosis relies on histopathology results, commonly presenting with DLBCL with the aid of immunohistochemistry[3]. Other histologies include a predominance of high-grade lymphomas with diffuse large B-cell, Burkitt, and Burkitt-like lymphoma[17].

Contrast-enhanced computed tomography (CECT) and double-contrast barium enema (DCBE) are the imaging modalities most commonly used to visualize the PCL, and they complement each other. CECT of the abdomen provides extraluminal information, such as tumor size, depth of invasion, and regional nodal involvement. However, DCBE provides more detailed information about mucosal changes and gross tumor morphology. It should be noted that neither of these modalities can differentiate between colonic carcinoma and lymphoma, and the diagnosis must be confirmed through colonoscopy and biopsy. Sub-classification requires immunohistochemistry[18]. FDG PET/CT scans have been shown to provide superior information compared with conventional imaging in gastrointestinal lymphomas, particularly for pre-treatment staging, post-treatment response evaluation, and, as highlighted in a study by Gupta *et al*, the detection of disease recurrence, thereby yielding results that are decisive for patient management^[^19,20^]^.

In clinical practice, the Lugano classification, based on modifications of the Ann Arbor system, is used for staging. The classification is based on the number of tumor sites (nodal and extranodal) and their location[8]. Furthermore, in extranodal DLBCL, the IPI and age-adapted IPI can be used for prognostic evaluation and differentiating between localized and advanced-stage disease[8]. Although we employed several suboptimal imaging modalities, we were still able to stage and risk-stratify the case effectively.

Systemic chemotherapy combined with immunotherapy is the primary treatment approach, with R-CHOP being the standard regimen for aggressive B-cell lymphomas^[^19,21^]^. A meta-analysis by Lightner *et al*, which included 28 studies (1658 patients) on non-gastric primary lymphoma, found that the majority of patients were treated with surgical resection followed by adjuvant chemotherapy, except for those with FL. The reason for this was that a large number of patients were diagnosed during surgery; 10–20% of patients required emergency surgery due to their clinical presentation. Regardless, the majority of authors concluded that a surgical approach followed by adjuvant chemotherapy is the correct course of treatment, resulting in the best overall survival. At the same time, a minority suggested that surgical excision alone or chemotherapy alone provides the best treatment outcomes. Moreover, several studies have shown that surgery has a positive impact on survival in multivariate analyses[21]. In contrast, FL, which is more prevalent in the duodenum, is treated differently because of its indolent course. Often, a strategy of watchful waiting is thought to be appropriate for patients with stage I disease. The majority of studies recommend surgery followed by adjuvant chemotherapy to prevent obstruction or perforation from the tumor for diseases in stages 2–4[21]. The patient in the index case report was initially treated with a right hemicolectomy, followed by systemic chemotherapy using the R-CHOP regimen.

Prognosis has markedly improved in recent decades, with current 5-year survival rates ranging from 60 to 80%, depending on the disease stage and histological subtype^[^21,22^]^. Poor prognostic factors include advanced stage, T-cell histology, older age, and the presence of B symptoms. However, localized disease, B-cell phenotype, and combined surgical and systemic therapy are associated with better outcomes^[^21,23^]^. The aim of our case report is to elaborate on the need for knowledge to recognize lymphoma in the colon for adolescents and its possibility of early management in relation to the case presentation. The strength of our case report is the availability of the data in relation to adequate history, investigation, and proper staging, as well as the appropriate management provided with adequate documentation. In addition, the long follow-up period strengthens the clinical value of the case report. However, the lack of imaging, such as endoscopy and a CT scan, has been a weakness, as we were unable to further elaborate on the presentation of the disease. Being a case report rather than a case series fails to provide a concrete elaboration of PCL presentations among adolescents.

## Conclusion

Primary lymphoma of the colon is a sporadic disease. Although it commonly occurs in an older age group (over 50), it can also occur atypically in younger patients, as in this case. Hence, it is essential to consider PCL as one of the differential diagnoses of colonic masses in young patients. The presentation is non-specific, leading to diagnosis at a late stage; hence, there is a crucial need for early diagnosis of PCL, which can improve outcomes through appropriate chemotherapy management that will enhance long-term results. Lastly, there is a need for more pediatric-specific data and long-term outcome studies in relation to PCL.

## Ethical approval

The case presentation was reviewed and approved by Ocean Road Research Review Comittee.

## Data Availability

The data presented in this manuscript are available from the authors.

## References

[1] Ghimire P Wu GY Zhu L. Primary gastrointestinal lymphoma. World J Gastroenterol 2011;17:697–707. 21390139 10.3748/wjg.v17.i6.697 PMC3042647

[2] Bairey O Ruchlemer R Shpilberg O. Non-Hodgkin’s lymphomas of the colon. Isr Med Assoc J 2006;8:832–35. 17214096

[3] Bandyopadhyay R Sinha SK Chatterjee U. Primary pediatric gastrointestinal lymphoma. Indian J Med Paediatr Oncol 2011;32. doi:10.4103/0971-5851.89786 PMC3237187 22174497

[4] Kassira N Pedroso FE Cheung MC. Primary gastrointestinal tract lymphoma in the pediatric patient: review of 265 patients from the SEER registry. J Pediatr Surg 2011;46:1956–64. 22008334 10.1016/j.jpedsurg.2011.06.006

[5] Ladd AP Grosfeld JL. Gastrointestinal tumors in children and adolescents. Semin Pediatr Surg 2006;15:37–47. 16458845 10.1053/j.sempedsurg.2005.11.007

[6] Goyal S. Primary colorectal lymphoma: a rare cause of small bowel obstruction. Ann Surg Case Reports & Images 2024;1:1–3.

[7] Skube SJ Arsoniadis EG Sulciner ML. Colorectal lymphoma: a contemporary case series. Dis Colon Rectum 2019;62:694–702. 30870226 10.1097/DCR.0000000000001373

[8] Aledavood A Nasiri MRG Memar B. Primary gastrointestinal lymphoma. J Res Med Sci 2012;17:487–90. 23626617 PMC3634278

[9] Green B Raman S. Colorectal lymphoma. Semin Colon Rectal Surg 2015;26:64–67.

[10] Dionigi G Annoni M Rovera F. Primary colorectal lymphomas: review of the literature. Surg Oncol 2007;16:169–71. 10.1016/j.suronc.2007.10.021 18024019

[11] Chen L Sun Q Chen E. Primary colonic lymphoma: report of two cases and a literature review. J Int Med Res 2021;49. doi:10.1177/03000605211017037 PMC8182193 34082600

[12] Kerwan A Al-Jabir A Mathew G. Revised Surgical Case Report (SCARE) guideline: an update for the age of Artificial Intelligence. Prem J Sci 2025. doi:10.70389/PJS.100079

[13] Jiao G Zheng Z Jiang K. Enteropathy-associated T-cell lymphoma presenting with gastrointestinal tract symptoms: a report of two cases and review of diagnostic challenges and clinicopathological correlation. Oncol Lett 2014;8:91–94. 24959225 10.3892/ol.2014.2105 PMC4063612

[14] Saka R Sasaki T Matsuda I. Chronic ileocolic intussusception due to transmural infiltration of diffuse large B cell lymphoma in a 14-year-old boy: a case report. Springerplus 2015;4:1–5. 26207197 10.1186/s40064-015-1157-6 PMC4508281

[15] Qadri SK Shah A Hamdani NH. Primary gastrointestinal lymphomas in children: an experience of 12 years from a tertiary care center of North India. Indian J Cancer 2016;53:300–03. 28071632 10.4103/0019-509X.197718

[16] Sangma MMB Dasiah SD Ashok AJ. Ileo-colic burkitt lymphoma in a young adult female - a case report. J ClinDiagn Res 2016;10:PD11–2. 10.7860/JCDR/2016/19105.7582 PMC4866183 27190885

[17] Pandey M Swain J Iyer HM. Primary lymphoma of the colon: report of two cases and review of literature. World J Surg Oncol 2019;17:1–7. 30646907 10.1186/s12957-018-1548-6 PMC6334463

[18] Kudaş İ. Colonic lymphoma presented as acute abdomen; A case report and review of literature. J Surg Med 2018;2:157–59.

[19] Duan Y Huang J Haybaeck J. Primary extranodal natural Killer/T-cell lymphoma in a child in the colon: a case report. Medicine (United States) 2021;100:E24232. 10.1097/MD.0000000000024232 PMC7837927 33546043

[20] Alotaibi SM Abdulrahman Misfer A Salma Hani Sait NMA. Colonic Burkitt’s lymphoma related to bowel obstruction in adults: a case report and literature review. Saudi Surg J 2020;8:148–51.

[21] Pande S Patne SC. Primary rectal lymphoma: a case report and review of literature. Indian J Nucl Med 2021;15:422–24. 10.4103/ijnm.ijnm_84_21 PMC8771074 35125760

[22] Ghazanfar H Jyala A Sun H. Diffuse large B-cell lymphoma in a young patient presenting as a cecal mass. Cureus 2022;14. doi:10.7759/cureus.31632 PMC9757662 36540509

[23] da Cunha Vasconcelos F de Castro Araujo RO Bernardo PS. Primary colorectal diffuse large B-cell lymphoma: a report of eighteen cases in a tertiary care center. Cancer Treat Res Commun 2023;36:1–6. 10.1016/j.ctarc.2023.100722 37331034

